# Measuring imagery strength in schizophrenia: no evidence of enhanced mental imagery priming

**DOI:** 10.1002/brb3.3146

**Published:** 2023-07-06

**Authors:** Sophie Wagner, Merlin Monzel

**Affiliations:** ^1^ Independent Researcher Dresden Germany; ^2^ Department of Psychology University of Bonn Bonn Germany

**Keywords:** binocular rivalry, mental imagery, schizophrenia, vividness of visual imagery

## Abstract

**Introduction:**

Recent research shows ambivalent results regarding the relationship between mental imagery and schizophrenia. The role of voluntary visual imagery in schizophrenic hallucinations remains unclear. The aim of the study was to investigate the association between visual imagery, schizophrenia, and the occurrence of schizophrenic hallucinations using an objective visual imagery task.

**Methods:**

The sample consisted of 16 participants with schizophrenia (59.1% female; *M*_Age_ = 45.55) and 44 participants without schizophrenia (62.5% female; *M*_Age_ = 43.94). Visual imagery was measured using the Vividness of Visual Imagery Questionnaire (VVIQ) as well as the well‐validated Binocular Rivalry Task (BRT). Occurrences of hallucinations were assessed using the Launay–Slade Hallucination Scale.

**Results:**

Participants with schizophrenia showed more hallucinatory experiences but did not score higher on either the VVIQ or the BRT than participants without schizophrenia. A correlation between the VVIQ and the BRT was found, validating the measurement of visual imagery and enabling the interpretation that visual imagery vividness is not enhanced in people with schizophrenia.

**Conclusion:**

The association between mental imagery vividness and schizophrenia found in previous studies may be based on other facets of mental imagery than visual imagery.

## Graphical abstract

The aim of the study was to investigate the association between visual imagery, schizophrenia, and the occurrence of schizophrenic hallucinations. Visual imagery was measured using the Vividness of Visual Imagery Questionnaire (VVIQ) and the well‐validated Binocular Rivalry Task (BRT). The results of the present study suggest that voluntary visual imagery is not directly linked to schizophrenia but moderates the association between these disorders and some types of hallucinations.

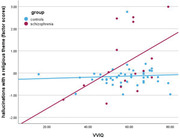

## 1 INTRODUCTION

The dopamine hypothesis of schizophrenia links an excessive activity of dopamine receptors with psychosis in schizophrenia. Morphologically, schizophrenia is characterized by changes in the brain, such as volume reductions in the primary visual cortex (Dorph‐Petersen et al., 2007), the frontal cortex (Shapleske et al., 2002), and the medial temporal lobe (Zipursky et al., 1994), including the hippocampus, amygdala, and insula (Bogerts et al., 1990; Rossi et al., 1994; Shapleske et al., 2002), as well as in regions around the ventricles (e.g., Andreasen et al., 1990). In addition, schizophrenia is linked to altered functional activity (Callicott et al., 2000; Manoach et al., 2000; Thermenos et al., 2005). These structural and functional changes can be accompanied by hallucinations (Cullberg & Nybäck, 1992; Flaum et al., 1995; Neckelmann et al., 2006; Suzuki et al., 1993), of whom visual hallucinations have a high prevalence in schizophrenia (Bracha et al., 1989). Visual hallucinations are in turn associated with functional changes in the brain similar to those seen in schizophrenia, for example, in the visual cortex (Braun et al., 2003; Ffytche et al., 1998).

Little explored in the context of schizophrenia and hallucinations is voluntary visual imagery, the inner sight in the absence of external sensory input (Kosslyn, 1995). Schizophrenia is associated with increased mental imagery (Sack et al., 2005); therefore, it is possible that the altered activation and volume reduction in different areas of the brain during schizophrenia and hallucinations lead to increased visual imagery, which could then in turn lead to more frequent and more severe hallucinations (Shine et al., 2015). This assumption arises because visual imagery activity largely corresponds to the aforementioned areas in the brain that underly morphological differences or altered activity in schizophrenia and hallucinations (Bergmann et al., 2016; Kreiman et al., 2000; Milton et al., 2021; Pearson, 2019; Sireteanu et al., 2008; Winlove et al., 2018). The volume reductions in schizophrenia might lead to an overactivation in the remaining parts of the affected structures, a compensatory mechanism, that is, for instance, also proposed for normal aging and Alzheimer's disease (Brodtmann et al., 2009; Johnson et al., 2000; Kalpouzos et al., 2012; Park & Reuter‐Lorenz, 2009). At the same time, dopaminergic overactivation and, in general, altered activity in the frontal cortex could lead to a loss of control of top‐down processes of visual imagery (Pearson, 2019), ultimately resulting in hallucinations and increased vivid imagery (Bergmann et al., 2016; Böcker et al., 2000; Mintz & Alpert, 1972).

Alternatively, since high visual imagery is linked to a higher frequency of hallucinations (Königsmark et al., 2021; Shine et al., 2015) and, on a cognitive level, visual worry (= “worrying by seeing something in the imagination”; Kowalski & Styła, 2022, p. 116) is a better predictor of positive psychotic symptoms than verbal worry (= “worrying with your inner speach”; Kowalski & Styła, 2022, p. 116), it could also be possible that visual imagery is associated with the occurrence of more frequent and more severe hallucinations in schizophrenia instead of being enhanced itself. For instance, Kowalski and Styła (2022) suggest aberrant salience, that is, an aberrant process of assigning attention to internal and external objects (Roiser et al., 2009), and biased source monitoring, that is, a biased attribution of percepts or memories to its perceived source (Brébion et al., 2000), as an explanatory account on the relationship between visual worry and positive psychotic symptoms. Thus, patients with schizophrenia might focus more on internally generated objects but misattribute their origin to an external source. Similarly, highly vivid imagery could also be misattributed to an external source (Dijkstra et al., 2021) and therefore be a risk factor for hallucinations in schizophrenia. Of note, hallucinations in schizophrenia are mainly auditory (Bauer et al., 2011), but visual hallucinations are the second most common hallucinations in schizophrenia, with a prevalence of 39.1%, and the methods of measuring visual imagery, as will be explained below, are more elaborate than the methods of measuring auditory imagery, which is why we chose to investigate visual imagery and visual hallucinations. However, visual imagery is highly correlated with auditory imagery (Betts, 1909; Jungmann et al., 2022), which is why visual imagery can be treated, at least to some extent, as a proxy for auditory imagery and its associations to auditory hallucinations.

### 1.1 Previous research

Previous research shows inconsistent findings regarding the relationship between schizophrenia and mental imagery. Using self‐report measures, some studies found no differences in mental imagery between individuals with schizophrenia and individuals without schizophrenia (Chandiramani & Varma, 1987; Matthews et al., 2014; Starker & Jolin, 1982), while others demonstrated that schizophrenia is associated with increased mental imagery (Oertel et al., 2009; Sack et al., 2005). Using experimental tasks to objectively measure visual imagery, Matthews et al. (2014) demonstrated that schizophrenia is associated with increased visual imagery ability by means of the Imagery Generation and Inspection Task (Zarrinpar et al., 2006). However, this evidence is very thin as the task involves spatial ability, which can be dissociated from object imagery ability (Blajenkova et al., 2006).

Exploring the differences in mental imagery between hallucinating and nonhallucinating patients, Starker and Jolin (1982) could not find differences in the vividness of imagery, Böcker et al. (2000) found decreased mental imagery in hallucinating patients, and, conversely, Aynsworth et al. (2017) found increased visual imagery in individuals with a high predisposition toward hallucinations. Thus, to enlighten these heterogenous findings, resolve the confounding of visual imagery measurement with spatial abilities, and examine the link to hallucinations, the present study applies a well‐validated version of the Binocular Rivalry Task (BRT; Keogh & Pearson, 2018; Pearson, 2014; Pearson et al., 2008, 2011) to measure visual imagery in participants with schizophrenia.

### 1.2 Advanced measurement of imagery strength via priming

Binocular rivalry is a process in which one image is presented to the left eye and another image is presented to the right eye at the same time, whereupon one of the images becomes dominant excluding the other image from awareness (Keogh & Pearson, 2018). Prior mental imagery of one of the two binocular rivalry patterns ends in a higher probability for that pattern to become dominant and conscious during the subsequent binocular rivalry display and can therefore prime subsequent binocular rivalry dominance (Pearson, 2014; Pearson et al., 2008, 2011). While Keogh and Pearson (2018) tested this paradigm successfully on people with aphantasia (= people without mental imagery), Shine et al. (2015) showed that it also produces reliable results on samples with Parkinson's disease and has therefore been proven to be applicable to samples that might deviate in cognitive or motor abilities.

### 1.3 Hypotheses

The purpose of the present study is to clarify whether there are differences in visual imagery between participants with schizophrenia (WHO, 2016) and participants without schizophrenia. Furthermore, it is to be examined whether these differences exist between hallucinating and nonhallucinating participants and whether increased imagery strength is associated with increased hallucinations in schizophrenia, either as a mediator or moderator.

## 2 METHOD

### 2.1 Participants

Participants without schizophrenia were acquired via social media groups. People with schizophrenic disorders were recruited from several psychiatric wards. A total of 66 participants were examined, including 22 participants with a disorder of the schizophrenia, schizotypal, and delusional disorder spectrum (WHO, 2016) and 44 participants without schizophrenia. Since the schizophrenia, schizotypal, and delusional disorder spectrum is rather heterogenous, we excluded participants with schizotypal and delusional disorders from our main analyses, but not from the validation analyses to provide higher power for the validation of measurements. The remaining 16 participants with schizophrenia were 37.5% male and 62.5% female and aged between 25 and 42 years (*M*_Age_ = 43.94, *SD*_Age_ = 11.86). The participants without schizophrenia were 56.8% male and 43.2% female and aged between 19 and 72 years (*M*_Age_ = 34.86, *SD*_Age_ = 16.76). Participants with schizophrenia had on average a significantly lower IQ score (*M* = 90.80, *SD* = 13.14) than participants without schizophrenia (*M* = 105.91, *SD* = 12.55) (*t*(58) = 4.07, *p* < .001, *d* = 1.19).

### 2.2 Measures

#### 2.2.1 Questionnaires

The Vividness of Visual Imagery Questionnaire (VVIQ) (Marks, 1973) was administered to assess subjective visual imagery. The occurrence and frequency of hallucinations were measured by the Launay–Slade Hallucination Scale—Revised (LSHS‐R; Waters et al., 2003). The mini‐q (Baudson & Preckel, 2016) captured the cognitive prerequisites of all participants. Finally, all participants answered some demographic questions, and participants with schizophrenia were questioned about their mental diagnosis according to ICD‐10 (WHO, 2016).

#### 2.2.2 Binocular Rivalry Task

Participants completed the BRT to measure their imagery strength, following the experimental design of Keogh and Pearson (2018). All participants were placed about 60 cm from the monitor and instructed to wear blue–red‐tinted anaglyph glasses to filter either red or blue light for the respective eye. At the beginning of the task, participants were once shown two Gabor patterns (1 cycle/°, Gaussian *σ* = 1.5°), one consisting of red‐horizontal lines (RGB: 255, 0, 0) and one consisting of blue‐vertical lines (RGB: 0, 0, 255). After that, two practice trials and 32 test trials were applied. In each trial, participants were presented with either the white letter R (for red) or B (for blue) in the middle of a black screen for 750 ms and asked to visualize the corresponding Gabor pattern that was shown at the beginning of the task. For visualization, the letters were followed by a black screen for 6000 ms. After that, participants were asked to indicate how vivid the visualization appeared in their mind's eye on a 4‐point Likert scale from *No image at all, you only “know” that you are thinking of the object* (1) to *Perfectly vivid* (4). This was followed by a Binocular Rivalry Stimulus for 750 ms, consisting of the aforementioned red‐horizontal and blue‐vertical Gabor patterns that were combined. Subsequently, participants were asked to indicate which type of lines they mainly saw during binocular rivalry, *blue‐vertical* (1), *perfectly mixed* (2), or *red‐horizontal* (3). Mock rivalry displays, either solid blue or solid red Gabor patterns, were presented in 12.5% of the trials. This was to detect whether participants performed the task correctly.

### 2.3 Procedure

Data were collected from May 19, 2021 to October 16, 2021 via the software PsychoPy v.2021.2.3 (Peirce, 2007, 2009) and SoSci Survey v.3.2.43 (Leiner, 2019). Inclusion criteria were age 18 years and older, computer skills, and proficiency in the German language. Before the experiment started, participants had to sign a privacy policy. Then, they were presented with items about demographics and, if in the experimental group, mental diagnosis according to ICD‐10 (WHO, 2016), followed by the VVIQ, the LSHS‐R, and the mini‐q. After the questionnaires, participants performed the BRT that included an eye dominance calibration according to Pearson et al. (2008).

### 2.4 Statistical analyses

In accordance with Keogh and Pearson (2018), the priming score of the BRT was computed by calculating the proportion of responses that matched the mental imagery cue. A sum score was calculated for the VVIQ. The LSHS‐R scores were determined by exploratory factor analysis to ensure that the same factor structure could be replicated in people with schizophrenia as in healthy individuals in whom the LSHS‐R had been validated previously (Waters et al., 2003). Reliability of measures was assessed via Cronbach's alpha for the VVIQ, the LSHS‐R, and the mini‐q and via split‐half correlation for the BRT. Convergent validity was assessed via heteromethod–monotrait correlation. Afterward, differences in imagery strength were assessed via *t*‐tests. Associations between schizophrenia and hallucinations as well as visual imagery and hallucinations were tested by means of *t*‐tests and correlations. Finally, to assess possible mediation and moderation effects of imagery strength on hallucinations, two competing models were tested via PROCESS v4.0 (Hayes, 2022) with the diagnosis group as independent variable, the LSHS‐R score as dependent variable, and the VVIQ score or BRT score as either mediator or moderator. Reanalyses of the data in which IQ scores were partialized out to control for group differences in intelligence yielded the same results as when intelligence was not considered.

## 3 RESULTS

### 3.1 Validation of measures

The exploratory factor analysis (Kaiser‐Meyer‐Olkin = .82, *χ* ^2^ = 501.46, *p* > .001) of the LSHS‐R revealed three factors (see Table 1), which could be interpreted as “vivid mental events,” “auditory and visual hallucinatory experiences,” and “hallucinations with religious theme,” thereby replicating the revised factor structure of the LSHS‐R by Waters et al. (2003).

**TABLE 1:** Factor structure of the Launay–Slade Hallucination Scale—Revised (LSHS‐R).

Item	M (*SD*)		Factor I	Factor II	Factor III
	Controls	Schizophrenia			
The people in my daydreams seem so true to life that sometimes I think they are	2.05 (1.14)	3.06 (1.44)	**.78**	.13	.22
Sometimes my thoughts seem as real as actual events in my life	2.91 (1.27)	3.56 (1.41)	**.67**	.09	.16
The sounds I hear in my daydreams are usually clear and distinct	2.23 (1.22)	3.56 (1.26)	**.66**	.10	.54
Sometimes a passing thought will seem so real that it frightens me	2.20 (1.21)	3.88 (1.31)	**.63**	.38	.06
No matter how hard I try to concentrate, unrelated thoughts always creep into my mind	2.61 (1.22)	3.56 (1.15)	**.51**	.35	–.16
In my daydreams I can hear the sound of a tune almost as clearly as if I were actually listening to it	2.52 (1.36)	3.56 (1.50)	**.48**	.05	.43
In the past I have had the experience of hearing a person's voice and then found that no one was there	1.39 (0.90)	2.94 (1.84)	.20	**.85**	.29
I have been troubled by hearing voices in my head	1.34 (0.89)	3.00 (1.79)	.08	**.81**	.27
On occasions I have seen a person's face in front of me when no one was in fact there	1.39 (0.97)	3.13 (1.78)	.34	**.50**	.47
I often hear a voice speaking my thoughts aloud	1.64 (1.10)	2.56 (1.71)	.28	**.46**	.21
I have heard the voice of the Devil	1.11 (0.62)	2.25 (1.73)	.11	.44	**.83**
In the past I have heard the voice of God speaking to me	1.23 (0.74)	2.44 (1.67)	.14	.47	**.75**
Eigenvalue			2.66	2.56	2.25
% of variance			22.16	21.37	18.79

*Note*. Factor loadings of items that have been assigned to a factor are printed in bold.

Reliabilities of all measures are depicted in Table 2 separated by diagnosis group. All measures show satisfactory reliability, excepting the BRT priming scores. Regarding convergent validity, a correlation between the VVIQ score and the priming score in the BRT was found (*r*(64) = .32, *p* = .009).

**TABLE 2:** Reliability measures for the Launay–Slade Hallucination Scale—Revised (LSHS‐R), the Vividness of Visual Imagery Questionnaire (VVIQ), the mini‐q, and the Binocular Rivalry Task depicted for each group.

Measure	Schizophrenia	Controls	Overall
LSHS‐R^a^	.82	.86	.89
Vivid mental events^a^	.66	.83	.83
Auditory and visual hallucinatory experiences^a^	.72	.83	.84
Hallucinations with a religious theme^a^	.98	.85	.96
VVIQ^a^	.90	.95	.93
mini‐q^a^	.94	.95	.96
Binocular Rivalry Task^b^	.22	.50	.33

^a^ Cronbach's alpha.

^b^ Split‐half reliability.

### 3.2 Group differences in visual imagery strength

No differences in imagery strength were found between participants with schizophrenia (VVIQ: *M* = 57.88, *SD* = 12.74; BRT: *M* = .53, *SD* = .06) and controls (VVIQ: *M* = 58.09, *SD* = 12.55; BRT: *M* = .55, *SD* = .12), neither in the VVIQ (*t*(58) = 0.06, *p* = .953, *d* = 0.02, 95% confidence interval [CI] [−0.56, 0.59], Bayes Factor (BF_01_) = 3.44) nor in the BRT (*t*(58) = 0.88, *p* = .384, *d* = 0.26, 95% CI [−0.32, 0.83], BF_01_ = 2.52). Analysis of mock trials revealed no evidence for incorrect response behavior, with the average priming score not being significantly different from chance, neither for participants with schizophrenia (*M* = .50, *SD* = .08) (*t*(15) = 0.05, *p* = .960, *d* = 0.01, 95% CI [−0.48, 0.50], BF_01_ = 3.91) nor for controls (*M* = .50, *SD* = .10) (*t*(43) = 0.26, *p* = .796, *d* = 0.04, 95% CI [−0.25, 0.33], BF_01_ = 5.93). When subdividing the patient group into those with hallucinations and those without hallucinations as in Shine et al. (2015), differences were still not significant (*p*s > .406). A post hoc power analyses based on the differences in imagery strength between Parkinson's disease patients with hallucinations and controls (*d* = 0.82; Shine et al., 2015) revealed a statistical power of 87% that is considered to be sufficient in clinical research studies (Suresh & Chandrashekara, 2012).

### 3.3 Associations with hallucinations

Participants with schizophrenia showed significantly higher scores on the LSHS‐R (*M* = 3.13, *SD* = 0.94) than participants without schizophrenia (*M* = 0.88, *SD* = 0.67) (*t*(20.80) = 4.85, *p* < .001, *d* = 1.66, 95% CI [1.00, 2.30], BF_10_ = 24,444.35). However, there were no significant correlations between the VVIQ and the LSHS‐R (*r*(58) = .15, *p* = .265, BF_01_ = 1.80) or between the BRT and the LSHS‐R (*r*(58) = –.07, *p* = .624, BF_01_ = 6.28).

### 3.4 Moderation and mediation analyses

**FIGURE 1:**
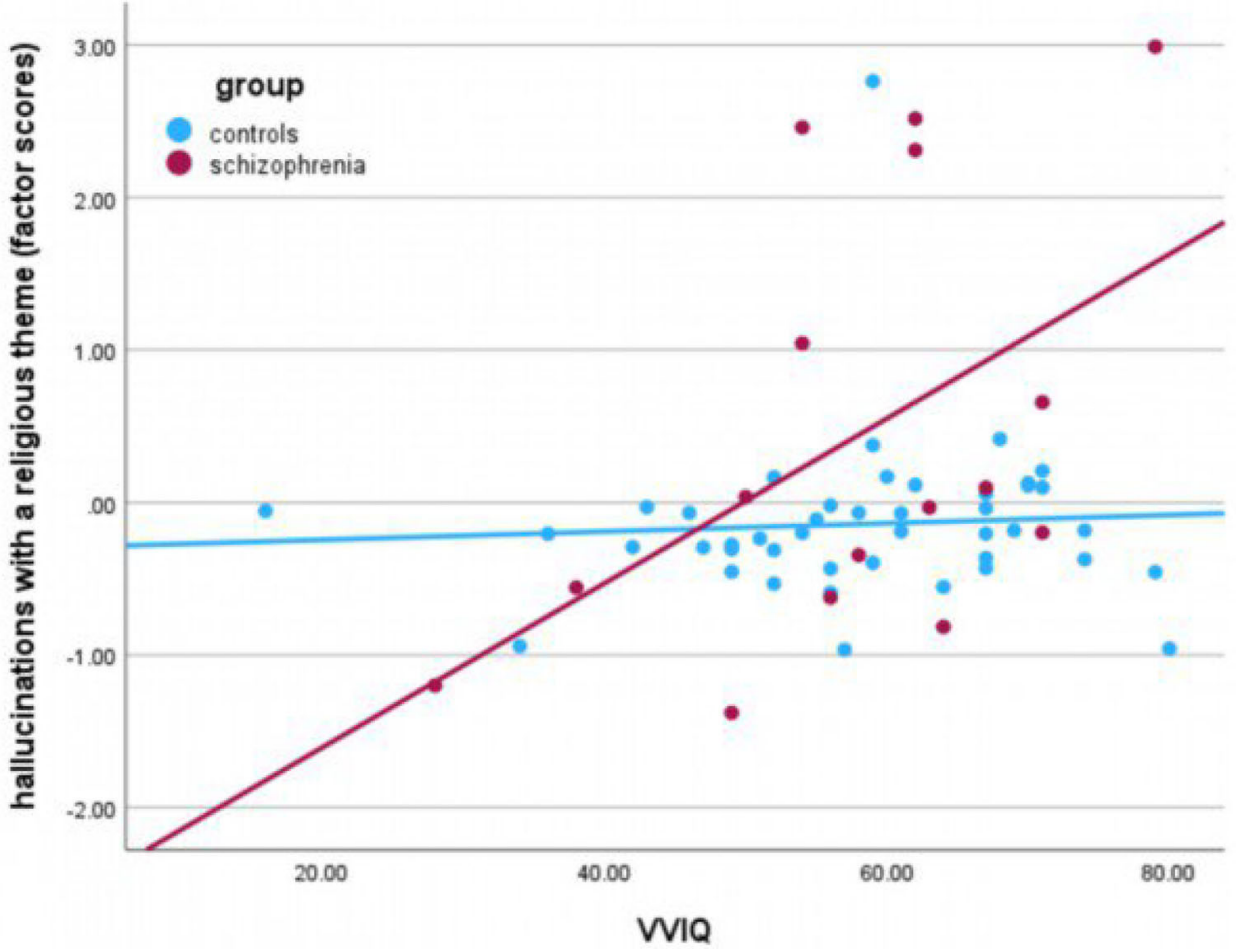
Moderation of the link between schizophrenia and religious hallucinations by vividness of visual imagery. Moderation effect between the Vividness of Visual Imagery Questionnaire (VVIQ) and group is significant (*F*(1, 56) = 7.35, *p* = .008, *R* ^2^ = .10, Bayes Factor (BF_10_) = 6.76). Slope coefficient for participants with schizophrenia is significant (*β* = .67, *p* = .002, 95% confidence interval [CI] [.26, .88]) but not for controls (*β* = .03, *p* = .781, 95% CI [–.27, .32]).

The link between schizophrenia and hallucinations was not moderated by visual imagery as measured by either the VVIQ (*F*(1, 56) = 0.26, *p* = .610, *R* ^2^ < .01, BF_01_ = 3.53) or the BRT (*F*(1, 56) = 0.67, *p* = .418, *R* ^2^ < .01, BF_01_ = 2.84). Likewise, there was no mediation between schizophrenia and hallucinations by visual imagery as measured by either the VVIQ (*β* = –.00, 95% CI [−0.12, 0.10]) or the BRT (*β* = –.00, 95% CI [−0.06, 0.05]). However, regarding the subscale “hallucinations with a religious theme,” a moderation by visual imagery measured via VVIQ could be found (*F*(1, 56) = 7.35, *p* = .008, *R* ^2^ = .10, BF_10_ = 6.76), providing significant slopes for participants with schizophrenia (*β* = .67, *p* = .002, 95% CI [0.27, 1.08]) but not for controls (*β* = .03, *p* = .781, 95% CI [−0.21, 0.28]) (see Figure 1).

## 4 DISCUSSION

Contrary to expectations, participants with schizophrenia did not score higher on either the VVIQ or the BRT than participants without schizophrenia. Thus, no significant group differences in visual imagery could be detected, although participants with schizophrenia showed higher scores on the LSHS‐R than participants without schizophrenia, which confirms their higher predisposition toward hallucinatory experiences. While there was a convergent correlation between the VVIQ and the BRT, the link between schizophrenia and hallucination was only moderated by visual imagery for the subscale “hallucinations with a religious theme.”

### 4.1 Appropriateness of measures

While all self‐report measures and the mini‐q exhibited appropriate reliability for both groups, the reliability of the BRT was relatively low due to low between‐subject variance (see Hedge et al., 2018). Besides, only 32 trials could be collected to ensure the reasonableness of the task. Reliability was even lower in participants with schizophrenia than in controls, which could be due to the even lower variance within the schizophrenic group or the cognitive impairments of participants with schizophrenia (Schaefer et al., 2013). However, the understanding of the task was successfully ensured by the test administration as no incorrect response behavior was detected in the mock trials. Overall, the BRT demonstrates convergent validity with the VVIQ, leading to the assumption that a reasonable degree of reliability must have prevailed despite the statistical limitations. Thus, the BRT extends the validity of its measure of visual imagery from samples with aphantasia (Keogh & Pearson, 2018) and Parkinson's disease (Shine et al., 2015) to samples consisting of participants with schizophrenia.

### 4.2 Visual imagery in schizophrenia

The results of the present study show no evidence for visual imagery being a trait marker of schizophrenia. This conclusion is reached using one of the currently best‐validated tasks to measure visual imagery, that is, the BRT, which does not have the aforementioned limitation of being confounded with spatial ability. Therefore, the argument that schizophrenia is associated with higher voluntary visual imagery is refuted. One explanation for the fact that voluntary visual imagery is not systematically increased in schizophrenia could be the large variability and individuality of morphological changes in the brain of individuals with schizophrenia (Seaton et al., 2001). Heterogenous changes could enhance voluntary visual imagery in some individuals (Oertel et al., 2009; Sack et al., 2005) or impede voluntary visual imagery in others (Böcker et al., 2000). On the other hand, both the BRT and the VVIQ do not measure the expression of involuntary visual imagery (e.g., hallucinations) and a direct inference from voluntarily imagery to involuntary imagery is not possible.

Regarding hallucinations, only a link between self‐reported visual imagery and “hallucinations with a religious theme” was found in the group with schizophrenia. This further evidences the assumption that visual imagery is not a consequence of schizophrenia, but that dispositional highly vivid imagery can exacerbate schizophrenia (cf. Shine et al., 2015). Hearing the voice of God or the devil is an extremely imaginative hallucination and might provoke distress, fear, and resistance (Chadwick & Birchwood, 1994) due to the hallucinatory perceptions and the omnipotence of the characters involved. However, the missing moderation effect for the other subscales might be confounded by hallucination modality, since it was not checked whether participants hallucinated predominantly visually or auditorily. While for the factor “hallucinations with a religious theme” this might not be a problem since many hallucinatory modalities are included, in the other factors effects might be diluted due to lack of congruence between imagery modality and hallucinatory modality. For instance, El Haj et al. (2019) and Shine et al. (2015) found increased visual imagery in individuals with schizophrenia only when they had visual hallucinations. The most frequent hallucinations in our sample were auditory (see Table 1), thus possibly explaining the missing moderation effect of imagery vividness between schizophrenia and the other factors of the LSHS‐R. However, the biggest difference between people with schizophrenia and controls was found for the item “On occasions I have seen a person's face in front of me when no one was in fact there,” which is clearly visual and therefore at least somewhat justifying our approach of using visual instead of other sensory imagery, which is only the second most common hallucinatory modality in schizophrenia (Bauer et al., 2011).

### 4.3 Implications

Since visual hallucinations might be aggravated in schizophrenia by dispositional visual imagery vividness, it could be seen as risk factor for a severe course of schizophrenia. An early assessment of visual imagery vividness in the psychological assessment of schizophrenia might therefore help to make the right choice of an appropriate therapeutic treatment. However, the moderation effect was rather small and based on a small sample size and should therefore be looked at more closely in future research. Previous studies have demonstrated that individuals with higher visual imagery experience more complex and vivid pseudohallucinations during the Ganzfeld technique (Königsmark et al., 2021) and more visual distortions during Pattern Glare (Dance et al., 2021), reinforcing the assumption that dispositional vividness of voluntary visual imagery might moderate the emergence of hallucinations. Using these methods in the future, involuntary visual imagery in schizophrenia could be examined more comprehensively. Regarding the missing direct link between visual imagery and schizophrenia, it cannot be excluded that only the occurrence of other sensory imagery might be enhanced in schizophrenia (Oertel et al., 2009; Sack et al., 2005). Thus, future research should focus on other sensory modalities of mental imagery in schizophrenia.

### 4.4 Limitations and appraisal

It has to be mentioned that all participants with schizophrenia were on medication. Antipsychotics result in a general sedation of cognitive processes (Herz et al., 1997), probably leading to difficulties in generating mental images within 6000 ms in the BRT. Ethically, however, medication cannot be withheld from participants. Nevertheless, while previous studies already showed correlations between the VVIQ and the BRT in nonclinical samples (Pearson et al., 2011), the present study was the first to apply the BRT to the measurement of visual imagery in schizophrenia that also contributes to the diagnostic performance assessment of the BRT. The BRT demonstrated convergent validity to the VVIQ in the present study even when participants were cognitively impaired and/or on medication. However, the sample of participants with schizophrenia was rather small and evidence for most null hypotheses according to Bayesian inference was only moderate (3 < BF_01_ < 10), so replication of the results should be sought in future studies.

## 5 CONCLUSIONS

Overall, the results of the present study suggest that voluntary visual imagery is not directly linked to schizophrenia but might moderate the association between schizophrenia and some types of hallucinations. It is therefore advisable to examine the associations of schizophrenia with different sensory modalities of voluntary and involuntary mental imagery more closely to detect trait markers that might predict the course and severity of schizophrenia.

### PEER REVIEW

The peer review history for this article is available at https://publons.com/publon/10.1002/brb3.3146.

## Data Availability

The data for all experiments are available at https://osf.io/czxvu/?view_only=0c4dd58a53c940c48f42a44d1db65721. None of the experiments was preregistered.
